# Combined Incisional Negative Pressure Wound Therapy and Subfascial Suction Drainage: The Suction Taco Approach

**DOI:** 10.7759/cureus.43577

**Published:** 2023-08-16

**Authors:** Matthew E Wells, Franklin J Powlan, Steven C Kieb, Nata Parnes, Andrew W Cleveland

**Affiliations:** 1 Orthopaedics and Rehabilitation, Texas Tech University Health Sciences Center El Paso, El Paso, USA; 2 Orthopaedic Surgery and Rehabilitation, Carthage Area Hospital, Carthage, USA; 3 Orthopaedic Surgery, Claxton-Hepburn Medical Center, Ogdensburg, USA; 4 Orthopaedics and Rehabilitation, William Beaumont Army Medical Center, El Paso, USA

**Keywords:** subfascial drain, taco technique, surgical site infection, posterior spinal fusion, wound care, fusion, spine surgery, spine, npwt, negative pressure wound therapy

## Abstract

An 18-year-old male with T4-L3 adult idiopathic scoliosis was treated with posterior spinal fusion followed by the application of a combined incisional negative pressure wound therapy (NPWT) and subfascial suction drainage system. In this report, we describe a novel technique that incorporates subfascial drains into an NPWT incisional vacuum system leading to a single exiting suction line. This effectively mitigates drain burden, maintains a sterile environment during the in-hospital postoperative period, provides NPWT to the drain exiting and incisional sites, and provides negative pressure-assisted deep space closure.

## Introduction

Surgical site infection (SSI) in spine surgery incurs significant patient morbidity, often leads to inferior outcomes, and imparts a substantial financial burden on the healthcare system. The incidence of spine SSI varies widely with non-instrumented procedures (i.e., laminectomy) displaying lower risks of 0-1% while instrumented fusions can have risks above 10% in the revision setting [1,2]. The readmission and treatment costs can range from $15,800 to $38,700 for adult deformity cases [3]. Therefore, preventing spine SSI is not only in the best interest of patients but also the financial state of the healthcare industry. 

There are several risk factors for spine-related SSI. Active smoking [4,5], poor long-term glycemic control [6,7], and elevated BMI [8,9] all serve as modifiable patient-related comorbidities that elevate the risk for SSI. Operational factors utilized to mitigate this risk include preoperative warming of patients, timely administration of antibiotics, skin preparation using chlorhexidine and alcohol solutions, meticulous surgical technique, maintenance of sterility, and appropriate closure techniques [10]. The placement of subfascial drains and the usage of incisional negative pressure wound therapy (NPWT) have become increasingly popular as adjunctive measures to prevent SSI following multi-level posterior spine surgery.

This report seeks to describe a case using a novel technique that incorporates subfascial drains into an NPWT wound vacuum-assisted closure (VAC) system (i.e. “taco technique”). This allows vacuum-assisted dead space closure, combined drain and incisional site NPWT, postoperative continuity of a sterile environment, and a decreased drain line burden during hospital recovery.

## Case presentation

A 14-year-old male Army dependent presented to the spine clinic after a referral from his primary care provider with the chief complaint of back deformity and pain. He was found to have an asymmetric Adam's forward bend test and was diagnosed with dextroscoliosis with a maximal Cobb angle of 37º on radiographic imaging. He was initially treated with thoracic-lumbar-sacral orthosis bracing, however, he was lost to follow-up due to multiple moves secondary to his father’s military occupation. He subsequently presented to our clinic at 18 years of age with a progression of his Cobb angle to 53º. Despite bracing and physical therapy, the patient endorsed persistent back pain and dissatisfaction with his appearance. The patient desired deformity correction and subsequently underwent T4-L3 posterior spinal fusion.

Technique

**Figure 1:**
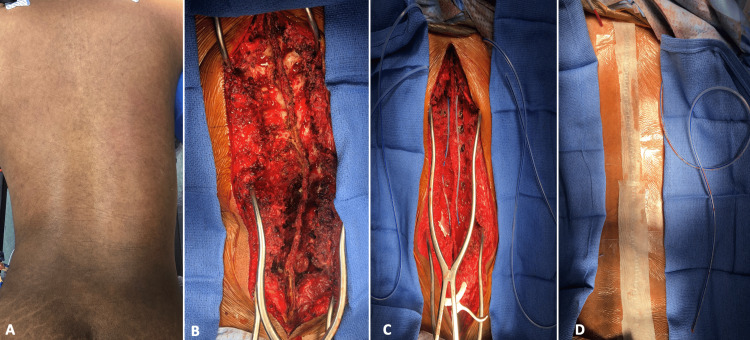
Initial steps to placement of the drain-incorporated, closed negative pressure incisional wound care system, “Taco technique”. (A) The operative field prior to prep and draping; (B) Surgical exposure utilizing an open posterior approach to the thoracolumbar spine; (C) Subfascial drains are placed deep to overlying fascia exiting proximally after completion of surgery; (D) Application of a nonadhesive dressing after formal wound closure with surrounding adhesive barrier strips

Once the operative intervention is completed (Figures 1A-1B), two subfascial percutaneous drains are placed on either side of the spinous process exiting percutaneously superior to the most proximal aspect of the surgical incision (Figure 1C). After the surgical incision is closed in a typical layered fashion, a non-adherent dressing (ADAPTIC™; 3M, Saint Paul, Minnesota, United States) is placed over the exposed surgical incision (Figure 1D). A liquid adhesive (Mastisol®, Eloquest Healthcare Inc., Ferndale, Michigan, United States) is applied in the peri-incisional area followed by an overlying border of adhesive dressing provided in the wound vacuum application dressing kit (Figure 1D). 

**Figure 2:**
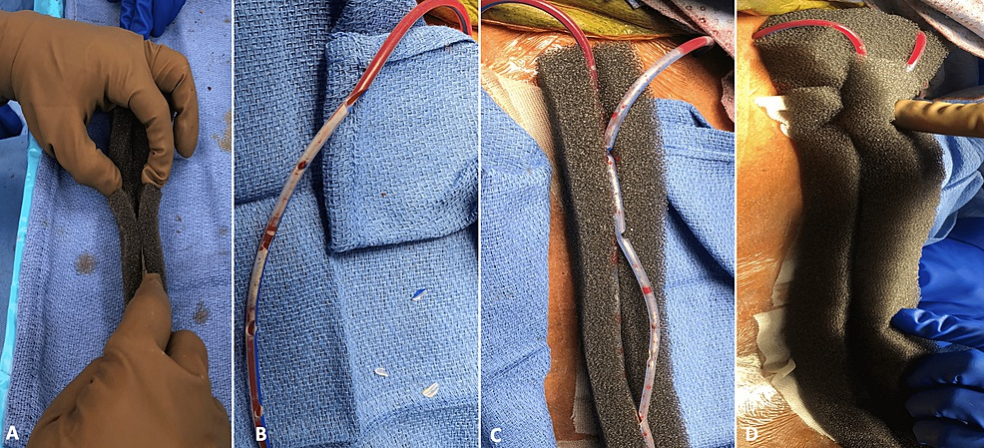
Preparation of the drains and incisional vacuum dressing followed by drain in-folding within the black sponge.

At this point, a wound VAC sponge is cut to the length and width of the incision. A partial thickness incision is then made along the middle of the length of the sponge to serve as a trough for eventual drain incorporation (Figure 2A). The drains are carefully prepared by perforating the drain tubes at regular intervals with suture scissors. (Figure 2B). The drains are then folded into the sponge trough, filling the sponge “taco” (Figure 2C). A second sponge is then cut to the same length and width to cover the incorporated drains (Figure 2D). 

**Figure 3:**
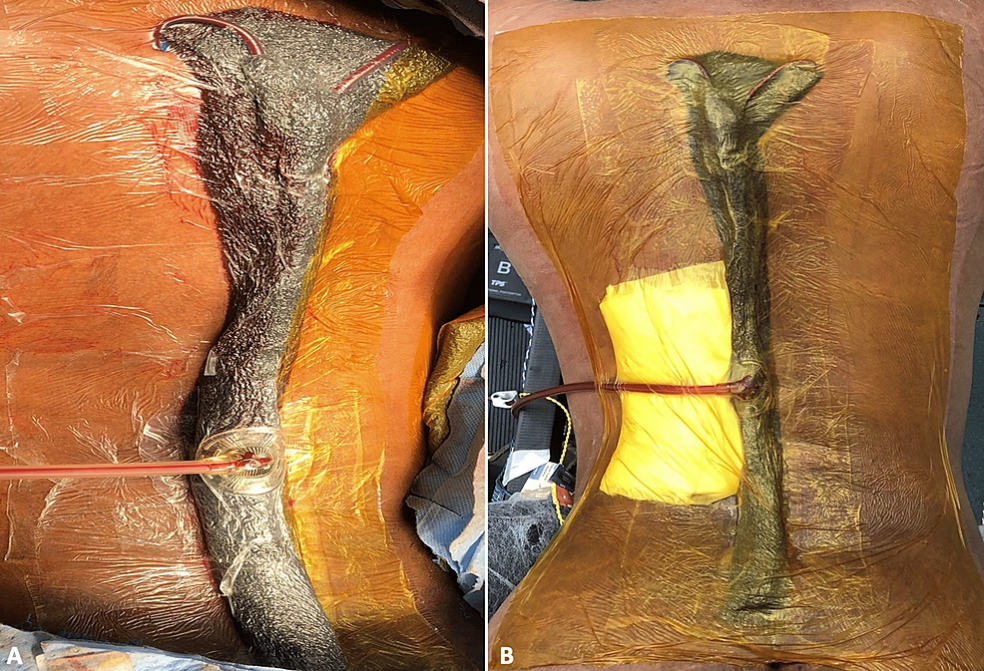
Final clinical imaging after applying the Taco postoperative wound care system.

Once the construct is completed, a provisional seal is obtained by applying the final adhesive dressing layer followed by lily pad application (Figure 3A). At this point, the surgeon can adjust the wound VAC to desired settings and confirm that the drains are working with the appropriate suction seal. We utilize 125 mmHg, medium continuous suction setting as extrapolated from a prior investigation [11]. After the seal check is completed, the exiting NPWT drain is padded to prevent underlying skin irritation, and the construct is further reinforced with an additional adhesive barrier as needed (Figure 3B). 

## Discussion

The use of NPWT and drains in multi-level posterior spine surgery remains standard practice, as both interventions have established benefits when utilized in the appropriate patient population. Incorporation of the subfascial drains into the NPWT incisional vacuum is a novel technique that provides vacuum-assisted dead space closure, provides combined drain and incisional site NPWT, allows for postoperative continuity of a sterile environment, and decreases drain line burden during inpatient recovery. This technique is simple, cost-effective, reproducible, and reliable.

The use of drains and NPWT has long been a standard of care in multi-level spine surgery. Subfascial drains may help prevent postoperative wound dehiscence via the egression of forming seromas/hematomas, minimize the need for wound aspirations, and potentially decrease the need for reoperation [12,13]. While prospective trials have shown equivalent outcomes, the decision to use or not use a wound drain following lumbar spine surgery should be left to the surgeon’s discretion [14]. Incisional NPWT has been shown to improve local tissue perfusion, improve oxygenation, reduce wound edge tension, provide a continued sterile closed environment, and can prevent dehiscence and SSI [15-17]. To our knowledge, the combined effect of subfascial drains in conjunction with NPWT has not been established and may provide an additive effect.

The ease of subfascial drain placement and the availability of NPWT with a relatively low cost (approximately $94 per day) in larger thoracolumbar reconstructive fusion surgery is considered prudent [18]. While the use of drains has been shown to increase the length of hospital stay, this has only been shown in shorter (≤ 3 levels) fusion constructs [18-20]. Conversely, drain use in extended multi-level posterior spinal fusions has shown lower rates of wound complication without a significant increase in transfusion requirement [13] Therefore, we recommend that this technique be considered in larger, multi-level posterior spinal fusion patients. These patients often remain in the hospital for several days postoperatively for pain control, physical therapy, and perioperative comorbidity management. It is the authors’ preference to transition patients to standard adhesive dressings prior to discharge. This ensures cost-effective care and negates patients’ burden of the NPWT machine when returning home.

## Conclusions

In this report, we described a novel technique that incorporates the subfascial drains into the NPWT incisional vacuum leading to a single exiting suction line. This effectively mitigates drain burden, maintains a sterile environment during the in-hospital stay, provides NPWT to the drain exiting and incisional sites, and provides negative pressure-assisted dead space closure. Further, this technique mitigates the risk of infection at drain sites, prevents suction drain failure secondary to clotting, permits more efficient dead space closure than bulb-assisted suction, and may lead to lower rates of postoperative seroma/hematoma formation, dehiscence, and possibly infection. Future comparative studies are required to objectively determine differences in rates of postoperative blood transfusions, dehiscence, and infections.
